# Asymptomatic Intracranial Arterial Stenosis and Metabolic Syndrome: The APAC Study

**DOI:** 10.1371/journal.pone.0113205

**Published:** 2014-12-02

**Authors:** Anxin Wang, Zhaoxia Li, Yanxia Luo, Xiaoxue Liu, Xiuhua Guo, Shouling Wu, Xingquan Zhao, Jost B. Jonas

**Affiliations:** 1 Department of Neurology, Beijing Tiantan Hospital, Capital Medical University, Beijing, China; 2 Department of Epidemiology and Health Statistics, School of Public Health, Capital Medical University, Beijing, China; 3 Beijing Municipal Key Laboratory of Clinical Epidemiology, Capital Medical University, Beijing, China; 4 Department of Cardiology, Tangshan People's Hospital, Tangshan, China; 5 Department of Cardiology, Kailuan Hospital, Hebei United University, Tangshan, China; 6 Department of Ophthalmology, Medical Faculty Mannheim of the Ruprecht-Karls-University of Heidelberg, Seegartenklinik Heidelberg, Germany; Fraunhofer Institute for Cell Therapy and Immunology, Germany

## Abstract

**Purpose:**

The metabolic syndrome (MetS) is a major risk factor for cardiovascular diseases. We investigated potential associations between MetS and asymptomatic intracranial arterial stenosis (ICAS) in a general population.

**Methods:**

The community-based “Asymptomatic Polyvascular Abnormalities in Community Study” examined asymptomatic polyvascular abnormalities in a Chinese population aged 40+ years without history of stroke and coronary heart disease. MetS was defined by the International Diabetes Federation criteria. Asymptomatic ICAS was diagnosed by transcranial color-coded Doppler sonography.

**Results:**

Out of 5393 study participants, asymptomatic ICAS was detected in 713 (13.2%) participants, and MetS in 1323 (24.5%) individuals. Prevalence of asymptomatic ICAS increased significantly from 7.5% to 24.2% with increasing number of MetS components. After adjusting for age, gender, physical activity, body mass index, low-density lipoprotein cholesterol and high-sensitivity C-reactive protein, MetS was significantly associated with asymptomatic ICAS (OR: 1.50; 95%CI: 1.23,1.83). Compared with the subgroup without MetS, the ORs for asymptomatic ICAS increased (*P*<0.0001) for each of 5 components of MetS from 1.71 (95%CI: 1.27,2.30), to 2.20 (95%CI: 1.63,2.98), 2.79 (95CI: 2.01,3.88), 3.08 (95%CI: 2.11,4.51) and 4.27 (95%CI: 2.22,8.20).

**Conclusions:**

In multivariate analysis, MetS was an independent and additional factor associated with asymptomatic ICAS. Study participants with 5 MetS components had a 4 times higher risk of asymptomatic ICAS than participants with no MetS component.

## Introduction

Intracranial arterial stenosis (ICAS) has been recognized as a serious cause of stroke, which is one of the worldwide leading causes of morbidity and mortality [1]–[3]. Previous studies demonstrated that the traditional vascular disease risk factors, including arterial hypertension, diabetes mellitus and hyperlipidemia were independently associated with ICAS in general and symptomatic populations [4]–[10].

The metabolic syndrome (MetS) is characterized by a cluster of risk factors, i.e., visceral obesity, dyslipidemia, hyperglycemia, and arterial hypertension [11]. These risk factors which are linked to insulin resistance and an increased risk of cardiovascular diseases have become major public-health challenges worldwide [12]–[18]. Previous hospital-based studies revealed that MetS is associated with an increased risk of symptomatic ICAS in patients with stroke [19]–[21]. Moreover, the proportion of ICAS increased with a higher number of MetS components. Despite of the high public health importance of MetS and of ICAS as major precursor of stroke and although addressing an asymptomatic stage as compared to treating the symptomatic stage of a disease is more effective and helpful, data are missing on the potential association between MetS and asymptomatic ICAS in a general population. We therefore conducted this study to investigate an association between MetS and asymptomatic ICAS in a community-based study population in which as compared to a hospital-based study population, the risk of a confounding effect by a bias due to the referral of patients may be less pronounced.

## Methods

The Asymptomatic Polyvascular Abnormalities Community study (APAC) is a community-based observational prospective, long-term follow-up study to investigate the epidemiology of asymptomatic polyvascular abnormalities in Chinese adults [22]. The study was performed according to the guidelines from the Helsinki Declaration and was approved by the Ethics Committees of the Kailuan General Hospital and the Beijing Tiantan Hospital. Written informed consent was obtained from all participants. Individuals were also informed of abnormal findings and recommended treatment. The study cohort was a sub-population of a previously described population of the Kailuan study which included a total of 101 510 employees and retirees of the Kailuan (Group) Co. Ltd, a large coal mine industry located in Tangshan, Hebei Province [23], [24]. The study population included mine workers as well as clerks with desk jobs. The city of Tangshan with approximately 7.2 million inhabitants in 2006 is situated 150 km southeast of Beijing and is a center of the coal mining industry. Using a stratified random sampling method by age and gender based on the data of the Chinese National Census from 2010, a sample of 7 000 subjects older than 40 years was randomly selected from the Kailuan cohort between June 2010 to June 2011. A total of 5 852 subjects agreed to participate in the APAC study and 5 816 people eventually completed the baseline data collection.

Among these 5,816 individuals, 376 subjects did not meet the inclusion criteria which were (1) no history of stroke, transient ischemic attack, and coronary disease at baseline as assessed by a validated questionnaire; and (2) absence of neurologic deficits indicating previous stroke as examined by experienced physicians. During the baseline survey, all participants underwent a clinical examination, laboratory tests, and carotid duplex ultrasound and transcranial Doppler examinations. Structured interviews with a standardized questionnaire were performed by trained investigators. The questionnaire included questions on the demographic and socioeconomic background, level of education, self-reported income, history of major medical disorders such as diabetes and hyperlipidemia, alcohol abuse and smoking. Anthropometric indices included height and weight. Body mass index (BMI) was calculated as body weight (kg) divided by the square of body height (m^2^). Smoking was defined as at least one cigarette per day for more than a year. Alcohol abuse was defined as alcohol intake of at least 90 g of liquor a day for more than one year for men and as alcohol intake of at least 45 g of liquor a day for more than one year for women. Smoking or drinking cessation was considered only if it lasted for at least one year. Details of the APAC study design and the information on baseline characteristics have been published previously [22]–[24].

MetS was defined using previously published criteria from the International Diabetes Federation [11]. The definition included the presence of central obesity (waist circumference ≥90 cm for Chinese men and ≥80 cm for Chinese women), plus any two of the following factors: triglycerides ≥150 mg/dL (1.7 mmol/L) or receiving specific treatment for elevated triglyceride concentrations; high-density lipoprotein cholesterol <40 mg/dL (1.03 mmol/L) in men and <50 mg/dL (1.29 mmol/L) in women, or receiving specific treatment for low high-density lipoprotein cholesterol; systolic blood pressure ≥130 mmHg or diastolic blood pressure ≥85 mmHg, or treatment of previously diagnosed arterial hypertension; fasting plasma glucose ≥100 mg/dL (5.6 mmol/L) or previously diagnosed type 2 diabetes mellitus.

Transcranial Doppler sonography was performed by two experienced neurologists using portable machines (EME Companion, Nicolet, Madison, WI, USA). Both neurologists when performing the sonography were unaware of the baseline information of the participants. Since they could fully see the study participants and thus estimate the BMI, they were not completely masked. Asymptomatic ICAS was diagnosed according to a peak systolic flow velocity of>140 cm/second in the middle cerebral artery,>120 cm/second in the anterior cerebral artery,>100 cm/second in the posterior cerebral artery and vertebra-basilar artery, and>120 cm/second in the siphon internal carotid artery [25]. In addition, the age of the patients, presence of disturbance in the echo frequency, turbulence, and whether the abnormal velocity was segmental were also taken into consideration for the diagnosis of ICAS. Patients were classified as having an occlusive disease if at least one of the studied arteries showed evidence of stenosis or occlusion.

Inclusion criteria for the present study were the availability of measurements of the blood concentration of triglycerides, high-density lipoproteins and glucose and of blood pressure. The statistical analysis was carried out using commercially available software (SAS software, version 9.3; SAS Institute Inc., Cary, NC, USA). We described continuous variables by their means ± standard deviations and categorical variables were described as percentages. We used the Student's t-test for non-paired samples for the comparison of normally distributed parameters and the Wilcoxon test for the comparison of non-parametric variables. The Chi-squared test was applied for the comparison of categorical variables. The trend of the prevalence of asymptomatic ICAS with increasing number of components of MetS was tested by the Chi-square trend test. We performed three multivariate logistic regression analyses to calculate odds ratios (OR) and 95% confidence intervals (CI) for the associations of MetS or the number of MetS components (the 0 MetS component group was used as the reference category) with asymptomatic ICAS. Model 1 adjusted for age and gender; Model 2 adjusted for age, gender, level of education, income, smoking, alcohol abuse, and physical activity; and Model 3 adjusted for age, gender, level of education, income, smoking, alcohol abuse, amount of physical activity, body mass index, and serum concentrations of low-density lipoprotein cholesterol and high-sensitivity C-reactive protein. Finally, for each model, a trend test was performed after the number of MetS components was entered into the model and treated as a continuous variable. We also used a multivariate stepwise logistic regression to analysis the independent associations between asymptomatic ICAS and MetS and all other variables in the model 3. Two-sided *P*-values were reported for all analyses. A *P*-value <0.05 was considered to be statistically significant. Subjects without a good temporal window were considered without stenosis. The statistical analysis was performed in two steps, either including the subjects without a good temporal window into the group of subjects without stenosis, or by excluding the subjects without a good temporal window from the statistical analysis. All data underlying the findings described in our manuscript are fully available without restrictions. Upon demand, the data will be send to and shared with any colleague interested.

## Results

Out of a total of 5440 subjects (2183 (40.1%) women) enrolled into the APAC study, individuals were excluded due to the non-availability of concentration measurements of triglycerides (n = 20 individuals) or high-density lipoprotein cholesterol (n = 3) or fasting plasma glucose (n = 20) or of blood pressure (n = 4). Eventually, 5,393 participants (3,230 men) with a mean age of 55.2±11.8 years (range: 40–94 years) were included into the present investigation. Some of the reasons for the non-availability were that subjects refused to get a venipuncture, or that the blood was not correctly sampled.

**Figure 1:**
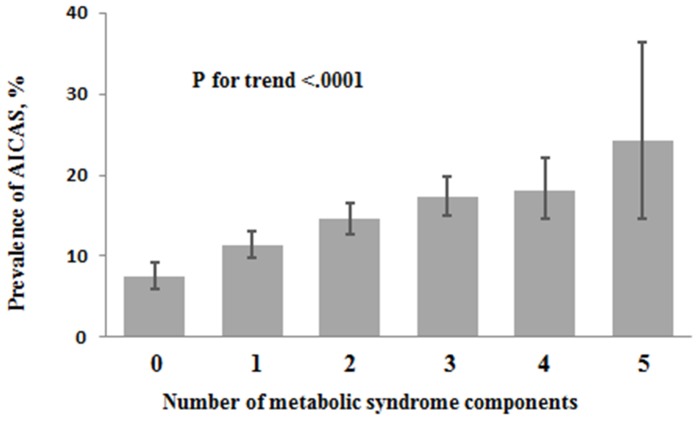
Prevalence (95% Confidence Interval) of Asymptomatic Intracranial Arterial Stenosis Stratified by the Number of Metabolic Syndrome Components in the Asymptomatic Polyvascular Abnormalities in Community Study (Unadjusted Data).

Asymptomatic ICAS was detected in 713 (13.2%) participants, and 1323 (24.5%) study participants had a MetS. The subgroup with asymptomatic ICAS compared to the subgroup without ICAS was significantly older (*P*<0.0001), had a lower level of education (*P*<0.0001), higher prevalence of former smoking (*P* = 0.0002) and alcohol abuse (*P* = 0.004), a higher prevalence of very active physical activity (*P* = 0.006), higher blood concentration of low-density lipoprotein (*P* = 0.002), high-density lipoprotein (*P* = 0.049), cholesterol (*P* = 0.049), high-sensitive C-reactive protein (*P*<0.001), and fasting plasma glucose (*P* = 0. 0001), longer waist circumference (*P* = 0.0007), and higher systolic blood pressure (*P*<0.0001). The frequency of central obesity, elevated arterial blood pressure, and of raised fasting plasma glucose concentration and the degree of MetS were significantly higher in the asymptomatic ICAS group than the group without ICAS (Table 1) (Fig. 1). The prevalence of asymptomatic ICAS increased significantly (*P*<0.0001) from 7.49% (95%CI: 5.96, 9.25) in the subgroup without any MetS component to 11.38% (95%CI: 9.79, 13.12) in the subgroup with one component of MetS, to 14.55% (95%CI: 12.77, 16.49) in the subgroup with two components, and to 24.24% (95%CI: 14.54, 36.36) in the subgroup with 5 components of MetS.

**Table 1:** Baseline Characteristics (Mean ± Standard Deviation), Frequency of Metabolic Syndrome and its Components, Stratified by the Presence of Asymptomatic Intracranial Arterial Stenosis, in the Asymptomatic Polyvascular Abnormalities in Community Study.

	Total	No Asymptomatic Intracranial Arterial Stenosis	Asymptomatic Intracranial Arterial Stenosis	*P*-Value
N	5393	4680	713	
Age (Years)	55.2±11.8	54.3±11.3	61.0±13.3	<0.0001
Male, n (%)	3230 (59.9)	2780 (59.4)	450 (63.1)	0.06
Education, n (%)				
Illiteracy/Primary School	658 (12.2)	505 (10.8)	153 (21.5)	<0.0001
Middle School	2373 (44.0)	2086 (44.6)	287 (40.3)	
High School or Higher	2362 (43.8)	2089 (44.6)	273 (38.3)	
Income, n (%)				
<¥1,000	1292 (24.0)	1121 (24.0)	171 (23.98)	0.24
¥1,000–3,000	3560 (66.0)	3102 (66.3)	458 (64.24)	
≥¥3,000	541 (10.0)	457 (9.8)	84 (11.78)	
Smoking, n (%)				
Never	3372 (62.5)	2946 (63.0)	426 (59.8)	0.0002
Former	303 (5.6)	239 (5.1)	64 (8.9)	
Current	1718 (31.9)	1495 (31.9)	223 (31.3)	
Alcohol abuse, n (%)	807 (14.96)	723 (15.45)	84 (11.78)	0.01
Physical Activity, n (%)				
Inactive	2159 (40.0)	1889 (40.4)	270 (37.9)	0.006
Moderately Active	1373 (25.5)	1213 (25.9)	160 (22.4)	
Very Active	1861 (34.5)	1578 (33.7)	283 (39.7)	
Body Mass Index (kg/m2)	24.9±3.3	24.9±3.3	25.01±3.1	0.36
Low-Density Lipoprotein-Cholesterol (mmol/L)	2.63±0.74	2.62±0.74	2.70±0.77	0.002
High-Sensitivity C-Reactive Protein	2.15±4.29	2.06±4.18	2.78±4.90	<0.0001
Waist Circumference (cm)	86.2±9.7	86.0±9.69	87.2±9.5	0.0007
Triglycerides (mmol/L)	1.68±1.41	1.68±1.43	1.66±1.28	0.15
High-Density Lipoprotein - Cholesterol (mmol/L)	1.63±0.45	1.63±0.46	1.60±0.43	0.049
Systolic Blood Pressure (mmHg)	131±20	130±19	142±221	<0.0001
Diastolic Blood Pressure (mmHg)	83±11	83±11	83±12	0.08
Fasting Plasma Glucose (mmol/L)	5.58±1.52	5.52±1.41	6.00±2.03	<0.0001
Metabolic Syndrome n (%)	1323 (24.5)	1091 (23.3)	232 (32.5)	<0.0001
Metabolic Syndrome Components				
Central Obesity, n (%)	2862 (53.1)	2456 (52.5)	406 (56.9)	0.03
Raised Triglycerides, n (%)	1759 (32.6)	1523 (32.5)	236 (33.1)	0.77
Reduced High-Density Lipoprotein - Cholesterol, n (%)	608 (11.3)	513 (11.0)	95 (13.3)	0.06
Raised Blood Pressure, n (%)	2271 (42.1)	1852 (39.6)	419 (58.8)	<0.0001
Raised Fasting Plasma Glucose, n (%)	1777 (33.0)	1456 (31.1)	321 (45.0)	<0.0001
Metabolic Syndrome (No. of Components)	1.72 ±1.26	1.67±1.25	2.07±1.24	<0.0001
0, n (%)	1042 (19.3)	964 (20.6)	78 (10.9)	<0.0001
1, n (%)	1459 (27.1)	1293 (27.6)	166 (23.)	
2, n (%)	1436 (26.6)	1227 (26.2)	209 (29.3)	
3, n (%)	944 (17.5)	781 (16.7)	163 (22.9)	
4, n (%)	446 (8.3)	365 (7.8)	81 (11.4)	
5, n (%)	66 (1.2)	50 (1.1)	16 (2.2)	

In the multivariate logistic regression analysis, with adjustment for age, gender, level of education, income, prevalence of smoking and alcohol abuse, amount of physical activity, body mass index, blood concentration of low-density lipoprotein, cholesterol and high-sensitivity C-reactive protein (model 3), MetS remained to be significantly associated with the prevalence of asymptomatic ICAS (OR: 1.50 (95%CI: 1.23, 1.83). Using the subgroup with no component of MetS as baseline, the ORs for the associations between the subgroups with 1, 2, 3, 4 and 5 MetS components and asymptomatic ICAS were 1.71 (95%CI: 1.27, 2.30), 2.20 (95%CI: 1.63, 2.98), 2.79 (95%CI: 2.01, 3.88), 3.08 (95%CI: 2.11, 4.51) and 4.27 (95%CI: 2.22, 8.20), respectively (model 3). In this multivariate model, the prevalence of asymptomatic ICAS increased significantly (*P* for trend <0.0001) with the number of MetS components. The same held true for the two other models of the multivariate analysis. The results in the participants with no good temporal window excluded was similar. (Table 2)

**Table 2:** Odds Ratios (95% Confidence Interval) of the Association between Metabolic Syndrome and Asymptomatic Intracranial Arterial Stenosis in the Asymptomatic Polyvascular Abnormalities in Community Study.

	Model 1: adjusted for age and gender;	Model 2: adjusted for age, gender, level of education, income, smoking, alcohol abuse, and amount of physical activity	Model 3: adjusted for age, gender, level of education, income, smoking, alcohol abuse, amount of physical activity, body mass index, and serum concentrations of low-density lipoprotein cholesterol and high-sensitivity C-reactive protein	Model 3: Participants with No Good Temporal Window Excluded
Metabolic Syndrome	1.56 (1.30–1.85)	1.54 (1.29–1.84)	1.50 (1.23–1.83)	1.50 (1.22–1.85)
No. of Components				
0	Reference	Reference	Reference	Reference
1	1.55 (1.16–2.06)	1.56 (1.17–2.09)	1.71 (1.27–2.30)	1.67 (1.23–2.25)
2	1.95 (1.48–2.58)	1.96 (1.48–2.59)	2.20 (1.63–2.98)	2.19 (1.62–2.97)
3	2.46 (1.84–3.28)	2.46 (1.84–3.30)	2.79 (2.01–3.88)	2.72 (1.95–3.80)
4	2.63 (1.87–3.69)	2.64 (1.87–3.71)	3.08 (2.11–4.51)	3.19 (2.17–4.71)
5	3.55 (1.91–6.59)	3.50 (1.87–6.55)	4.27 (2.22–8.20)	4.19 (2.14–8.23)
*P* for trend	<0.0001	<0.0001	<0.0001	<0.0001

In the multivariate stepwise logistic regression including all potential variables in model 3, in addition to MetS (OR: 1.49; 95%CI: 1.25, 1.78), asymptomatic ICAS was associated with a lower level of education and with a higher concentration of low-density lipoprotein cholesterol (Table 3).

**Table 3:** Multivariate Stepwise Logistic Regression Analysis for Associations with Asymptomatic Intracranial Arterial Stenosis in the Asymptomatic Polyvascular Abnormalities in Community Study.

	Odds Ratio (95% Confidence Interval)	*P*-Value
Metabolic Syndrome	1.49 (1.25–1.78)	<0.0001
Age, year	1.04 (1.03–1.05)	<0.0001
Level of education		
Illiteracy/Primary School	Reference	
Middle School	0.71 (0.56–0.90)	0.0002
High School or Higher	0.64 (0.51–0.82)	0.0039
Low-Density Lipoprotein Cholesterol, mmol/L	1.12 (1.01–1.25)	0.029

## Discussion

In our community-based study population of 5,393 individuals, MetS defined by the new definitions of the International Diabetes Federation was significantly associated with asymptomatic ICAS, independently of additional risk factors for ICAS such as age, male gender, lower level of education, smoking, higher body mass index and blood concentration of lipids. The prevalence of asymptomatic ICAS increased significantly and linearly from 7.5% to 24.2% with increasing number of MetS components. Study participants with 5 MetS components had a 4 times higher risk of asymptomatic ICAS than participants with no MetS component. As the first community-based study reporting on the association of MetS with asymptomatic ICAS in a general population, our investigation confirms previous hospital-based studies which as hospital-based investigations were mostly focused on symptomatic ICAS.

Previous hospital-based studies suggested that MetS was independently associated with symptomatic ICAS in stroke population [19]–[21], [26]–[29]. An association between MetS and asymptomatic ICAS was previously reported by Mi and colleagues who prospectively examined effect of MetS on the prognosis of ischemic stroke secondary to intracranial stenosis in Chinese patients [27]. In a group of 701 patients with ischemic stroke due to ICAS, MetS was identified in 26% of the patients. In multivariate Cox proportional hazards analysis with adjustment for gender, BMI, smoking, diabetes, and low-density lipoproteins, the 1-year stroke recurrence was significantly associated with the presence of MetS (hazard ratio 2.30; 95% CI: 1.01–5.22) and large waist circumference (hazard ratio: 2.39; 95% CI: 1.05–5.42). Park and associates assessed whether the apolipoprotein B to apolipoprotein A ratio (apoB)/apoAI ratio) was more closely associated with an ICAS or an extracranial arterial stenosis [27]. In a sample of 464 patients with acute ischemic stroke, the subgroup with ICAS (n = 236) showed a higher apoB/apoAI ratio (0.81±0.02) than both the subgroup with extracranial atherosclerotic stenosis (n = 44) (0.74±0.03) and the subgroup with no cerebral atherosclerotic stenosis (n = 184) (0.72±0.02) groups (P = 0.002). The ratio was substantially increased (0.93±0.03) in patients with advanced ICAS (≥3 intracranial stenoses). With a multivariable analysis, the highest apoB/apoAI ratio quartile was an independent predictor of ICAS (OR, 2.13; 95% CI, 1.05 to 4.33). The authors concluded that a higher apoB/apoAI ratio was a predictor of ICAS rather than of extracranial atherosclerotic stenosis or no cerebral atherosclerotic stenosis. In an investigation by Park *et al.*, 378 acute ischemic stroke patients underwent brain magnetic resonance imaging and magnetic resonance angiography [20]. Thicker carotid intimal medial thickness values were associated a higher number of MetS components (*P*<0.001). MetS was independently associated with intracranial atherosclerosis (OR: 3.58; 95%CI: 2.28–5.63), which was prominent with more severe MetS components after adjustment for other risk factors (*P*<0.001). Bang and collaborators evaluated whether MetS was associated with intracranial atherosclerotic stroke [29]. In a study population of 439 patients with ischemic stroke or transient ischemic attacks, MetS was observed more frequently in patients with intracranial atherosclerosis than in those with other types of stroke (*P* = 0.003). In a multiple regression analysis, metabolic syndrome, but not conventional risk factors, was independently associated with intracranial atherosclerosis (*P* = 0.02). Ding *et al.* performed a meta-analysis to evaluate the different influence of parameters such as sex, arterial hypertension, diabetes, dyslipidemia, smoking, age and MetS on ICAS compared to extracranial arterial stenosis in Asian population, by searching PUBMED, EMBASE and Web of Science databases [29]. They found that patients with MetS were more likely to suffer from ICAS than from extracranial arterial stenosis, with a pooled OR of ICAS versus ECAS of 1.68 (95%CI: 1.32–2.12; *P*<0.0001).

All the preceding studies were hospital-based investigations in contrast to our study with a community-based study population. In addition, most of the preceding studies were focused on symptomatic ICAS while our investigation addressed asymptomatic ICAS. From a practical point of view, knowledge of associations of asymptomatic ICAS may be as least as important as knowledge of associations of symptomatic ICAS, since prophylactic measures have more impact in taken in the asymptomatic stage.

Our result of the association between MetS and asymptomatic ICAS warrants prospective studies to assess whether management of MetS and its components will improve the prognosis of asymptomatic ICAS, in particular whether it will lead to a decreased risk of eventual stroke. Management of MetS may include drug therapy, modification of risk factors, surveillance, and other procedures. To cite an example, in the next follow-up investigations of the Kailuan study one may address whether study participants who underwent a successful drug therapy of MetS or who profoundly changed their lifestyle and nutrition as compared to individuals with MetS but without effective therapy will show a reduced incidence of asymptomatic ICAS and a reduced frequency of a conversion of asymptomatic ICAS into symptomatic IACS including stroke. Similar studies may also be performed in Europe, since associations between MetS and stenosis of the carotid arteries may depend on the ethnic background and on differences in lifestyle and nutrition.

Potential limitations of our study should be mentioned. First, a cross-sectional study as ours cannot prove a causal relationship which usually has to be shown in a longitudinal investigation. The results of our study only allow concluding on an association between MetS and asymptomatic ICAS, while the role of MetS as a risk factor for asymptomatic ICAS may be shown in follow-up investigations. Second, transcranial Doppler sonography was used to diagnose asymptomatic ICAS without confirmation of the result by magnetic resonance angiography or other forms of angiography. However, transcranial Doppler sonography is a widely accepted method for screening intracranial occlusive diseases, especially in a general population [30]. Third, though we used a stratified random sampling method to minimize inclusion bias, residual confounding could not entirely be excluded. Our study was based on a randomly selected subgroup of the participants of the large Kailuan Study which included employees and retirees of the Kailuan Company. One may argue that despite its large study sample, the Kailuan Study population may not have been representative for the population of the city of Tangshan or the province of Hebei. The study population was selected using a stratified random sampling method by age and gender based on the data of the Chinese National Census from 2010. It is however, likely that mine workers differ from workers in other professions. A sampling method based on age and gender alone might have accounted for this.

In conclusion, our community-based study with a more than 5000 study participants revealed that MetS was significantly associated with asymptomatic ICAS, independently of additional risk factors for ICAS. The prevalence of asymptomatic ICAS increased linearly from 7.5% to 24.2% with increasing number of MetS components, so that individuals with 5 MetS components had a 4 times higher risk of asymptomatic ICAS than participants with no MetS component. Future studies may prospectively assess whether therapy of MetS in the asymptomatic stage of ICAS can help to reduce the risk of eventual stroke.
